# Supplementary material for: Nonlinear association between stress hyperglycemia ratio and severe consciousness disorder in acute ischemic stroke: A MIMIC retrospective analysis
Source: PLoS One. 2025 Aug 21;20(8):e0329678. doi: 10.1371/journal.pone.0329678 (PMC12370082; doi:10.1371/journal.pone.0329678)
Supplement: S1 Table — Data are expressed as mean (SD), median (Q1-Q3) or N (%). Abbreviations: BMI (body mass index), ALT(alanine aminotransferase), AST(aspartate aminotransferase), BUN (blood urea nitrogen), HbA1c (hemoglobin a1c), AF(Atrial Fibrillation), CHD(coronary heart disease), CKD(chronic kidney disease), HF(heart failure), RF(respiratory failure), SHR(stress hyperglycemia ratio) (DOCX) [file pone.0329678.s001.docx]

**S1 Table. Baseline characteristics of the GCS>8 and GCS≤8 groups**

| **Parameters** | **GCS>8** | **GCS≤8** | **P-value** |
| --- | --- | --- | --- |
|  | **N=3484** | **N=307** |  |
| Age, years | 73 (61-83) | 73 (63.50-84) | 0.231 |
| BUN,mg/dl | 17.00 (13.00-22.00) | 19.00 (14.00-25.50) | <0.001 |
| Serum creatinine，mg/dL | 0.90 (0.70-1.10) | 1.00 (0.80-1.30) | 0.006 |
| Hemoglobin,g/dL | 12.49 (1.97) | 12.08 (2.07) | <0.001 |
| Bicarbonate,mEq/L | 24.93 (3.25) | 23.41 (3.89) | <0.001 |
| Potassium,mEq/L | 4.05 (0.50) | 4.15 (0.73) | <0.001 |
| Chloride,mEq/L | 103.52 (4.15) | 103.76 (4.75) | 0.344 |
| White blood cell, k/uL | 7.90 (6.20-10.10) | 10.40 (7.80-13.50) | <0.001 |
| Platelet ,k/uL | 217.00 (176.00-267.00) | 206.00 (160.00-268.50) | 0.018 |
| Total cholesterol,mg/dL | 170.37 (47.54) | 160.97 (51.29) | <0.001 |
| Blood glucose,mg/dL | 107.00 (94.00-137.00) | 131.00 (107.00-168.00) | <0.001 |
| SHR, mg/dl | 0.89 (0.78-1.04) | 1.03 (0.86-1.19) | <0.001 |
| HBA1C,% | 6.30 (1.46) | 6.40 (1.45) | 0.225 |
| Gender |  |  | 0.972 |
| Male | 1744 (50.06%) | 154 (50.16%) |  |
| Female | 1740 (49.94%) | 153 (49.84%) |  |
| Marital status |  |  | <0.001 |
| Married | 1620 (46.50%) | 123 (40.07%) |  |
| Single | 783 (22.47%) | 64 (20.85%) |  |
| Widowed | 628 (18.03%) | 44 (14.33%) |  |
| Divorced | 238 (6.83%) | 12 (3.91%) |  |
| Missing | 215 (6.17%) | 64 (20.85%) |  |
| Race |  |  | <0.001 |
| White | 2397 (68.80%) | 170 (55.37%) |  |
| Other | 506 (14.52%) | 94 (30.62%) |  |
| Black | 490 (14.06%) | 34 (11.07%) |  |
| Asian | 91 (2.61%) | 9 (2.93%) |  |
| BMI |  |  | 0.005 |
| Underweight | 41 (1.18%) | 4 (1.30%) |  |
| Normal | 355 (10.19%) | 30 (9.77%) |  |
| Overweight | 506 (14.52%) | 45 (14.66%) |  |
| Obese | 792 (22.73%) | 98 (31.92%) |  |
| Missing | 1790 (51.38%) | 130 (42.35%) |  |
| Lymphocyte, k/uL |  |  | <0.001 |
| <1.2 | 188 (5.40%) | 35 (11.40%) |  |
| 1.2-1.7 | 306 (8.78%) | 32 (10.42%) |  |
| >1.7 | 14 (0.40%) | 2 (0.65%) |  |
| Missing | 2976 (85.42%) | 238 (77.52%) |  |
| Neutrophil,k/uL |  |  | <0.001 |
| <1.6 | 18 (0.52%) | 1 (0.33%) |  |
| 1.6-6.1 | 229 (6.57%) | 15 (4.89%) |  |
| 6.1-8.6 | 124 (3.56%) | 19 (6.19%) |  |
| >8.6 | 137 (3.93%) | 34 (11.07%) |  |
| Missing | 2976 (85.42%) | 238 (77.52%) |  |
| Albumin,g/dl |  |  | <0.001 |
| <3.8 | 788 (22.62%) | 117 (38.11%) |  |
| ≥3.8 | 667 (19.14%) | 58 (18.89%) |  |
| Missing | 2029 (58.24%) | 132 (43.00%) |  |
| ALT,iu/l |  |  | <0.001 |
| <40 | 1793 (51.46%) | 182 (59.28%) |  |
| ≥40 | 197 (5.65%) | 46 (14.98%) |  |
| Missing | 1494 (42.88%) | 79 (25.73%) |  |
| AST,iu/l |  |  | <0.001 |
| ＜40 | 1751 (50.26%) | 160 (52.12%) |  |
| ≥40 | 250 (7.18%) | 70 (22.80%) |  |
| Missing | 1483 (42.57%) | 77 (25.08%) |  |
| Creatine kinase,iu/l |  |  | <0.001 |
| <47 | 296 (8.50%) | 30 (9.77%) |  |
| 47-322 | 1310 (37.60%) | 109 (35.50%) |  |
| >322 | 218 (6.26%) | 49 (15.96%) |  |
| Missing | 1660 (47.65%) | 119 (38.76%) |  |
| Creatine kinase-MB,ng/mL |  |  | <0.001 |
| <3.0 | 1092 (31.34%) | 95 (30.94%) |  |
| 3.0-10.0 | 512 (14.70%) | 54 (17.59%) |  |
| >10.0 | 93 (2.67%) | 37 (12.05%) |  |
| Missing | 1787 (51.29%) | 121 (39.41%) |  |
| Total bilirubin,mg/dL |  |  | <0.001 |
| ＜1.5 | 1779 (51.06%) | 204 (66.45%) |  |
| ≥1.5 | 65 (1.87%) | 9 (2.93%) |  |
| Missing | 1640 (47.07%) | 94 (30.62%) |  |
| AF | 724 (20.78%) | 96 (31.27%) | <0.001 |
| Alcohol use | 42 (1.21%) | 5 (1.63%) | 0.521 |
| Anemia | 443 (12.72%) | 98 (31.92%) | <0.001 |
| Cancer | 257 (7.38%) | 26 (8.47%) | 0.485 |
| CHD | 911 (26.15%) | 105 (34.20%) | 0.002 |
| CKD | 276 (7.92%) | 27 (8.79%) | 0.589 |
| Diabetes mellitus | 1139 (32.69%) | 118 (38.44%) | 0.04 |
| HF | 564 (16.19%) | 95 (30.94%) | <0.001 |
| Hypertension | 1387 (39.81%) | 128 (41.69%) | 0.518 |
| Hyperlipidemia | 1290 (37.03%) | 91 (29.64%) | 0.01 |
| RF | 179 (5.14%) | 124 (40.39%) | <0.001 |
| Tobacco use | 256 (7.35%) | 20 (6.51%) | 0.59 |
| Anticoagulant drugs | 393 (11.28%) | 105 (34.20%) | <0.001 |
| Antiplatelet drugs | 889 (25.52%) | 131 (42.67%) | <0.001 |
| Long-term follow-up time,days | 14.89 (3.13-339.62) | 20.20 (8.66-61.38) | 0.244 |
| long-term mortality |  |  | <0.001 |
| No | 2576 (73.94%) | 125 (40.72%) |  |
| Yes | 908 (26.06%) | 182 (59.28%) |  |

**S2 Table：Shapiro-Wilk test**

| Variable | W Statistic | P Value |
| --- | --- | --- |
| Age | 0.9664 | < 2.2e-16 |
| BUN | 0.7537 | < 2.2e-16 |
| Chloride | 0.9766 | < 2.2e-16 |
| Creatine kinase | 0.4051 | < 2.2e-16 |
| Blood glucose | 0.724 | < 2.2e-16 |
| Hemoglobin | 0.9899 | 7.86E-16 |
| HbA1c | 0.7401 | < 2.2e-16 |
| Bicarbonate | 0.9812 | < 2.2e-16 |
| Potassium | 0.9263 | < 2.2e-16 |
| PLT | 0.8725 | < 2.2e-16 |
| Total cholesterol | 0.9549 | < 2.2e-16 |
| Triglyceride | 0.5332 | < 2.2e-16 |
| White blood cell | 0.5652 | < 2.2e-16 |

**S3 Table：Statistical Analysis of Continuous Variables Using Dunn's Test with Bonferroni Correction Across SHR Tertiles**

| Variable | High vs. Low | High vs. Medium | Low vs. Medium |
| --- | --- | --- | --- |
| Age | Z = -1.86, ns | Z = 0.99, ns | Z = 2.86, ** |
| Blood urea nitrogen (BUN) | Z = 3.02, ** | Z = 5.07, *** | Z = 2.05, ns |
| Chloride | Z = -6.98, ** | Z = -5.49, *** | Z = 1.49, ns |
| Creatine kinase | Z = 1.40, ns | Z = 3.75, *** | Z = 2.35, * |
| Blood glucose | Z = 40.00, ** | Z = 24.07, *** | Z = -15.93, *** |
| Hemoglobin | Z = 0.34, ns | Z = -4.33, *** | Z = -4.67, *** |
| HbA1c | Z = -7.68, ** | Z = 3.45, *** | Z = 11.13, *** |
| Bicarbonate | Z = -8.71, ** | Z = -6.38, *** | Z = 2.34, * |
| Potassium | Z = 1.55, ns | Z = 1.42, ns | Z = -0.14, ns |
| Platelet | Z = -3.61, ** | Z = -1.55, ns | Z = 2.06, ns |
| Total cholesterol | Z = -0.68, ns | Z = -4.37, *** | Z = -3.69, *** |
| Triglyceride | Z = 2.40, * | Z = 2.28, * | Z = -0.12, ns |
| White blood cell | Z = 12.74, ** | Z = 9.01, *** | Z = -3.73, *** |
|  | | | |

Statistical comparisons were performed using the Mann-Whitney U test. P adj < 0.001 is indicated by "***"; P adj < 0.01 is indicated by "**"; P adj < 0.05 is indicated by "*"; ns = not significant.

**S4 Table: Statistical Evaluation of Categorical Variables by SHR Tertiles Using Chi-Square and Fisher's Exact Tests with FDR Correction**

| **Variable** | **Low vs Medium** | **Low vs High** | **Medium vs High** |
| --- | --- | --- | --- |
| Gender | p=0.030 * | p=0.233 ns | p=0.349 ns |
| BMI | p=0.073 ns | p=0.041 * | p=0.533 ns |
| Marital Status | p=0.043 * | p=0.000 *** | p=0.001 ** |
| Race | p=0.000 *** | p=0.000 *** | p=0.010 ** |
| Albumin | p=0.000 *** | p=0.004 ** | p=0.000 *** |
| ALT | p=0.316 ns | p=0.001 *** | p=0.061 ns |
| AST | p=0.092 ns | p=0.000 *** | p=0.011 * |
| Creatine Kinase | p=0.017 * | p=0.000 *** | p=0.149 ns |
| Creatine Kinase-MB | p=0.019 * | p=0.000 *** | p=0.002 ** |
| Lymphocyte | p=0.282 ns | p=0.000 *** | p=0.000 *** |
| Neutrophil | p=0.382 ns | p=0.000 *** | p=0.000 *** |
| Total Bilirubin | p=0.000 *** | p=0.000 *** | p=0.430 ns |
| AF | p=0.776 ns | p=0.138 ns | p=0.069 ns |
| Anemia | p=0.350 ns | p=0.000 *** | p=0.000 *** |
| Cancer | p=0.934 ns | p=0.452 ns | p=0.553 ns |
| CHD | p=0.050 * | p=0.064 ns | p=0.000 *** |
| CKD | p=0.608 ns | p=0.942 ns | p=0.511 ns |
| DM | p=0.000 *** | p=0.000 *** | p=0.000 *** |
| HF | p=0.002 ** | p=0.006 ** | p=0.000 *** |
| Hypertension | p=0.606 ns | p=0.000 *** | p=0.002 ** |
| RF | p=0.479 ns | p=0.000 *** | p=0.000 *** |
| Tobacco Use | p=0.878 ns | p=0.071 ns | p=0.042 * |

All statistical comparisons were conducted using chi-square tests or Fisher's exact tests (depending on the data characteristics). P adj < 0.001 is indicated by "***"; P adj < 0.01 is indicated by "**"; P adj < 0.05 is indicated by "*"; ns = not significant.
