# Supplementary material for: Nonlinear association between stress hyperglycemia ratio and severe consciousness disorder in acute ischemic stroke: A MIMIC retrospective analysis
Source: PLoS One. 2025 Aug 21;20(8):e0329678. doi: 10.1371/journal.pone.0329678 (PMC12370082; doi:10.1371/journal.pone.0329678)
Supplement: Attachment — Submitted filename: Response to Reviewers.docx [file pone.0329678.s005.docx]

Dear Editor and Reviewers,

Thank you for your thorough review and valuable feedback on my manuscript titled “[**Nonlinear association between stress hyperglycemia ratio and severe consciousness disorder in acute ischemic stroke: A MIMIC retrospective analysis**]” (Manuscript ID: **PONE-D-24-59138**). I have taken all comments seriously and revised the manuscript according to the reviewers' suggestions. Below are detailed responses to each comment.

Reviewer #1:

The topic discussed is very interesting and innovative for the management and prognosis of patients with stroke. Although I must make some small suggestions.
General:
1. You should not use too many abbreviations or acronyms, even less so if the term is only one word. This makes it difficult to read.

Thank you for your review and feedback on our manuscript. Regarding your comment about “the excessive use of abbreviations and acronyms, especially when the term is only one word,” we sincerely understand and value this point. To address your concern, we have made the following adjustments:

**Response:**

1) **Original Text (Lines 65-67)**:

In recent years, the stress hyperglycemia ratio has garnered significant attention in the prognostic assessment of patients with acute ischemic stroke.

**Revised Text**:

In recent years, the SHR has garnered significant attention in the prognostic assessment of patients with AIS.

2) **Original Text (Lines 102-114)**:

Demographic information included age, sex, race, marital status, and body mass index (BMI).Laboratory parameters included white blood cell (WBC) count, lymphocyte count, neutrophil count, platelet count, hemoglobin (HB), albumin (ALB), alanine aminotransferase (ALT), aspartate aminotransferase (AST), total bilirubin (TBIL), blood urea nitrogen (BUN), serum creatinine (SCR), creatine kinase (CK), creatine kinase-MB (CKMB), blood glucose (GLU), glycated hemoglobin A1c (HbA1c), bicarbonate(HCO3), chloride, potassium, total cholesterol(TC), and triglyceride (TG). Comorbidities: identified using ICD-9 or ICD-10 codes, including hypertension (HBP), diabetes mellitus (DM), hyperlipidemia (HL), anemia, cancer, atrial fibrillation (AF), coronary artery disease (CAD), chronic kidney disease (CKD), respiratory failure (RF), heart failure (HF), and history of alcohol use or smoking.

**Revised Text**:

Demographic information included age, sex, race, marital status, and body mass index (BMI). Laboratory parameters included the following: white blood cell count, lymphocyte count, neutrophil count, platelet count, hemoglobin, albumin, alanine aminotransferase (ALT), aspartate aminotransferase (AST), total bilirubin, blood urea nitrogen (BUN), serum creatinine, creatine kinase, creatine kinase-MB, blood glucose, glycated hemoglobin A1c (HbA1c), bicarbonate, chloride, potassium, total cholesterol, and triglycerides. Comorbidities were identified using International Classification of Diseases, Ninth Revision (ICD-9) codes or Tenth Revision (ICD-10) codes, including hypertension, diabetes mellitus, hyperlipidemia, anemia, cancer, atrial fibrillation (AF), coronary artery disease (CAD), chronic kidney disease (CKD), respiratory failure (RF), heart failure (HF), and a history of alcohol use or smoking.

3) **Original Text (Lines 144-149)**:

This model included all variables from Model 2 plus ALB, ALT, BUN, CK, CKMB, chloride, SCR, HB, HCO3, potassium, lymphocyte count, platelet count, TC, triglycerides, WBC, AF, alcohol use, anemia, cancer, CAD, CKD, DM, HF, HBP, HL, RF, tobacco use, anticoagulant drugs, and antiplatelet drugs.

**Revised Text**:

This model included all variables from Model 2 plus albumin, ALT, BUN, creatine kinase, creatine kinase-MB, chloride, serum creatinine, hemoglobin, bicarbonate, potassium, lymphocyte count, platelet count, total cholesterol, triglycerides, white blood cell count, AF, alcohol use, anemia, cancer, CAD, CKD, DM, HF, hypertension, hyperlipidemia, RF, tobacco use, anticoagulant drugs, and antiplatelet drugs.

4) **Original Text (Lines 179-186)**:

Abbreviations: BMI (body mass index), ALB(album), ALT(alanine aminotransferase), AST(aspartate aminotransferase), BUN (blood urea nitrogen), CK( creatine kinase), CKMB(creatine kinase lsoenzyme MB), SCR (serum creatinine), GLU(glucose), HB(hemoglobin), HbA1c (hemoglobin a1c), TBIL(Total Bilirubin), TC (total cholesterol), TG (triglyceride), WBC((white blood cell), AF(Atrial Fibrillation), CHD(coronary heart disease), CKD(chronic kidney disease), DM(Diabetes Mellitus), HF(heart failure), HBP(high blood pressure), HL(hyperlipidemia), RF(respiratory failure), SHR(stress hyperglycemia ratio)

**Revised Text**:

Abbreviations: BMI (body mass index), ALB(album), ALT(alanine aminotransferase), AST(aspartate aminotransferase), BUN (blood urea nitrogen), HbA1c (hemoglobin a1c), AF(Atrial Fibrillation), CHD(coronary heart disease), CKD(chronic kidney disease), HF(heart failure), RF(respiratory failure), SHR(stress hyperglycemia ratio)

5) **Original Text (Lines 212-215)**:

Model 3 was adjusted Model2 and ALB, ALT, BUN, CK, CKMB, Chloride, SCR, HB, HCO3, Potassium, Lymphocyte, Platelet, TC, Triglyceride, WBC, AF, Alcohol use, Anemia, Cancer, CHD, CKD, DM, HF, HBP, HL, RF, Tobacco use, Anticoagulant drugs, Antiplatelet drugs. HR (Hazard Ratio), CI (Confidence Interval)

**Revised Text**:

Model 3 was adjusted Model2 and albumin, ALT, BUN, creatine kinase, creatine kinase-MB, Chloride, serum creatinine, hemoglobin, bicarbonate, Potassium, Lymphocyte, Platelet, total cholesterol, triglyceride, white blood cell, AF, Alcohol use, Anemia, Cancer, CHD, CKD, DM, HF, hypertension, hyperlipidemia, RF, Tobacco use, Anticoagulant drugs, Antiplatelet drugs. HR (Hazard Ratio), CI (Confidence Interval)

6) **Original Text (Lines 245-248)**:

Model 3 was adjusted for the variables in model 2 and further adjusted for ALB, ALT, BUN, CK, CKMB, Chloride, SCR, HB, HCO3, Potassium, Lymphocyte, Platelet, TC, Triglyceride, WBC, AF, Alcohol use, Anemia, Cancer, CHD, CKD, DM, HF, HBP, HL, RF, Tobacco use, Anticoagulant drugs, Antiplatelet drugs.

**Revised Text**:

Model 3 was adjusted for the variables in model 2 and further adjusted for albumin, ALT, BUN, creatine kinase, creatine kinase-MB, Chloride, serum creatinine, hemoglobin, bicarbonate, Potassium, Lymphocyte, Platelet, total cholesterol, Triglyceride, white blood cell, AF, Alcohol use, Anemia, Cancer, CHD, CKD, DM, HF, hypertension, hyperlipidemia, RF, Tobacco use, Anticoagulant drugs, Antiplatelet drugs.

7) **Original Text (Lines 261-264)**:

**Fig 4. Forest plots of stratified analyses of SHR and outcomes.** a: Forest plot of HRs for GCS≤8 in different subgroups. b: Forest plots of HRs for long-term mortality in different subgroups. DM (diabetes mellitus), AF (Atrial Fibrillation), HBP(high blood pressure)

**Revised Text**:

**Fig 4. Forest plots of stratified analyses of SHR and outcomes.** a: Forest plot of HRs for GCS≤8 in different subgroups. b: Forest plots of HRs for long-term mortality in different subgroups. diabetes mellitus, AF (Atrial Fibrillation), hypertension

8) **Original Text (Lines 503-509)**:

Data are expressed as mean (SD), median (Q1-Q3) or N (%). Abbreviations: BMI (body mass index), ALB(album), ALT(alanine aminotransferase), AST(aspartate aminotransferase), BUN (blood urea nitrogen), CK( creatine kinase), CKMB(creatine kinase lsoenzyme MB), SCR (serum creatinine), GLU(glucose), HB(hemoglobin), HbA1c (hemoglobin a1c), TBIL(Total Bilirubin), TC (total cholesterol), TG (triglyceride), WBC((white blood cell), AF(Atrial Fibrillation), CHD(coronary heart disease), CKD(chronic kidney disease), DM(Diabetes Mellitus), HF(heart failure), HBP(high blood pressure), HL(hyperlipidemia), RF(respiratory failure), SHR(stress hyperglycemia ratio)

**Revised Text**:

Data are expressed as mean (SD), median (Q1-Q3) or N (%). Abbreviations: BMI (body mass index), ALT(alanine aminotransferase), AST(aspartate aminotransferase), BUN (blood urea nitrogen), HbA1c (hemoglobin a1c), AF(Atrial Fibrillation), CHD(coronary heart disease), CKD(chronic kidney disease), HF(heart failure), RF(respiratory failure), SHR(stress hyperglycemia ratio)

9) **Original Text (Line 177 Table 1. Baseline characteristics by SHR tertiles N=3791)**:

| **Parameters** | **Low** | **Medium** | **High** | **P-value** |
| --- | --- | --- | --- | --- |
|  | N=1264 | N=1263 | N=1264 |  |
| Age, years | 71.96 (14.75) | 70.42 (14.78) | 70.91 (14.88) | 0.028 |
| BUN,mg/dl | 17.00 (13.00-22.00) | 16.00 (12.00-21.00) | 18.00(13.00-24.00) | <0.001 |
| Chloride,mEq/L | 104.02 (4.15) | 103.72 (3.96) | 102.89 (4.40) | <0.001 |
| SCR,mg/dL | 0.90 (0.80-1.20) | 0.90 (0.70-1.10) | 0.90 (0.80-1.20) | <0.001 |
| GLU,mg/dL | 99.75 (28.57) | 114.91 (32.52) | 166.90 (71.41) | <0.001 |
| HB,g/dL | 12.37 (1.88) | 12.69 (1.92) | 12.34 (2.11) | <0.001 |
| HBA1C% | 6.57 (1.63) | 6.07 (1.23) | 6.28 (1.44) | <0.001 |
| HCO3,mEq/L | 25.28 (3.45) | 24.99 (2.97) | 24.14 (3.44) | <0.001 |
| Potassium,mEq/L | 4.03 (0.47) | 4.04 (0.50) | 4.09 (0.58) | 0.004 |
| Platelet ,k/uL | 233.95 (83.39) | 228.40 (86.29) | 223.79 (89.85) | 0.013 |
| TC,mg/dL | 167.74 (44.22) | 173.15 (43.73) | 167.23 (49.63) | 0.002 |
| Triglyceride,mg/dL | 110.00 (80.00-146.00) | 108.00 (81.00-147.00) | 114.05 (83.00-154.00) | 0.026 |
| WBC, k/uL | 7.40 (6.00-9.20) | 7.80 (6.30-9.90) | 9.00 (6.90-11.90) | <0.001 |
| Gender |  |  |  | 0.086 |
| Male | 604 (47.78%) | 659 (52.18%) | 635 (50.24%) |  |
| Female | 660 (52.22%) | 604 (47.82%) | 629 (49.76%) |  |
| BMI |  |  |  | 0.067 |
| <23.9 | 158 (12.50%) | 131 (10.37%) | 136 (10.76%) |  |
| 23.9-27.9 | 205 (16.22%) | 174 (13.78%) | 177 (14.00%) |  |
| >27.9 | 271 (21.44%) | 296 (23.44%) | 323 (25.55%) |  |
| Missing | 630 (49.84%) | 662 (52.41%) | 628 (49.68%) |  |
| Marital status |  |  |  | <0.001 |
| Married | 588 (46.52%) | 598 (47.35%) | 557 (44.07%) |  |
| Single | 270 (21.36%) | 279 (22.09%) | 298 (23.58%) |  |
| Widowed | 248 (19.62%) | 212 (16.79%) | 212 (16.77%) |  |
| Divorced | 101 (7.99%) | 88 (6.97%) | 61 (4.83%) |  |
| Missing | 57 (4.51%) | 86 (6.81%) | 136 (10.76%) |  |
| Race |  |  |  | <0.001 |
| White | 840 (66.46%) | 894 (70.78%) | 833 (65.90%) |  |
| Other | 164 (12.97%) | 189 (14.96%) | 247 (19.54%) |  |
| Black | 222 (17.56%) | 145 (11.48%) | 157 (12.42%) |  |
| Asian | 38 (3.01%) | 35 (2.77%) | 27 (2.14%) |  |
| ALB,g/dl |  |  |  | <0.001 |
| <3.8 | 261 (20.65%) | 207 (16.39%) | 292 (23.10%) |  |
| >=3.8 | 247 (19.54%) | 325 (25.73%) | 298 (23.58%) |  |
| Missing | 756 (59.81%) | 731 (57.88%) | 674 (53.32%) |  |
| ALT,iu/l |  |  |  | 0.004 |
| <18 | 360 (28.48%) | 351 (27.79%) | 327 (25.87%) |  |
| >=18 | 352 (27.85%) | 386 (30.56%) | 442 (34.97%) |  |
| Missing | 552 (43.67%) | 526 (41.65%) | 495 (39.16%) |  |
| AST,iu/l |  |  |  | <0.001 |
| <22 | 370 (29.27%) | 342 (27.08%) | 306 (24.21%) |  |
| >=22 | 348 (27.53%) | 397 (31.43%) | 468 (37.03%) |  |
| Missing | 546 (43.20%) | 524 (41.49%) | 490 (38.77%) |  |
| CK,iu/l |  |  |  | <0.001 |
| 47-322 | 452 (35.76%) | 494 (39.11%) | 490 (38.77%) |  |
| <47 | 115 (9.10%) | 92 (7.28%) | 102 (8.07%) |  |
| >322 | 63 (4.98%) | 88 (6.97%) | 116 (9.18%) |  |
| Missing | 634 (50.16%) | 589 (46.63%) | 556 (43.99%) |  |
| CKMB,ng/mL |  |  |  | <0.001 |
| <3 | 271 (21.44%) | 237 (18.79%) | 233 (18.43%) |  |
| 3.0-10 | 317 (25.08%) | 354 (28.07%) | 339 (26.82%) |  |
| >10 | 19 (1.50%) | 36 (2.85%) | 75 (5.93%) |  |
| Missing | 657 (51.98%) | 634 (50.28%) | 617 (48.81%) |  |
| Lymphocyte, k/uL |  |  |  | <0.001 |
| <1.08 | 39 (3.09%) | 55 (4.35%) | 95 (7.52%) |  |
| 1.08-1.74 | 58 (4.59%) | 57 (4.51%) | 85 (6.72%) |  |
| >1.74 | 62 (4.91%) | 51 (4.04%) | 75 (5.93%) |  |
| Missing | 1105 (87.42%) | 1100 (87.09%) | 1009 (79.83%) |  |
| Neutrophil,k/uL |  |  |  | <0.001 |
| <5.1 | 64 (5.06%) | 59 (4.67%) | 66 (5.22%) |  |
| 5.1-8.1 | 65 (5.14%) | 60 (4.75%) | 72 (5.70%) |  |
| >8.1 | 30 (2.37%) | 44 (3.48%) | 117 (9.26%) |  |
| Missing | 1105 (87.42%) | 1100 (87.09%) | 1009 (79.83%) |  |
| TBIL,mg/dL |  |  |  | <0.001 |
| <0.4 | 164 (12.97%) | 133 (10.53%) | 150 (11.87%) |  |
| 0.4-0.7 | 371 (29.35%) | 360 (28.50%) | 372 (29.43%) |  |
| >0.7 | 121 (9.57%) | 188 (14.89%) | 198 (15.66%) |  |
| Missing | 608 (48.10%) | 582 (46.08%) | 544 (43.04%) |  |
| AF | 265 (20.97%) | 258 (20.43%) | 297 (23.50%) | 0.135 |
| Alcohol use | 13 (1.03%) | 22 (1.74%) | 12 (0.95%) | 0.14 |
| Anemia | 151 (11.95%) | 135 (10.69%) | 255 (20.17%) | <0.001 |
| Cancer | 90 (7.12%) | 92 (7.28%) | 101 (7.99%) | 0.676 |
| CAD | 339 (26.82%) | 295 (23.36%) | 382 (30.22%) | <0.001 |
| CKD | 103 (8.15%) | 95 (7.52%) | 105 (8.31%) | 0.744 |
| DM | 412 (32.59%) | 329 (26.05%) | 516 (40.82%) | <0.001 |
| HF | 220 (17.41%) | 163 (12.91%) | 276 (21.84%) | <0.001 |
| HBP | 495 (39.16%) | 501 (39.67%) | 519 (41.06%) | 0.601 |
| HL | 495 (39.16%) | 481 (38.08%) | 405 (32.04%) | <0.001 |
| RF | 64 (5.06%) | 73 (5.78%) | 166 (13.13%) | <0.001 |
| Tobacco use | 99 (7.83%) | 102 (8.08%) | 75 (5.93%) | 0.076 |
| Anticoagulant drugs | 156 (12.34%) | 158 (12.51%) | 184 (14.56%) | 0.186 |
| Antiplatelet drugs | 352 (27.85%) | 353 (27.95%) | 315 (24.92%) | 0.149 |
| Follow-up time for GCS ≤ 8,days | 3.67 (2.08-5.78) | 3.74 (2.33-6.04) | 4.78 (2.70-8.33) | <0.001 |
| GCS ≤ 8 |  |  |  | <0.001 |
| No | 1205 (95.33%) | 1191 (94.30%) | 1088 (86.08%) |  |
| Yes | 59 (4.67%) | 72 (5.70%) | 176 (13.92%) |  |
| Long-term follow-up time,days | 22.52 (3.09-520.34) | 11.35 (3.00-240.25) | 16.39 (4.23-199.28) | 0.005 |
| Long-term mortality |  |  |  | <0.001 |
| No | 912 (72.15%) | 974 (77.12%) | 815 (64.48%) |  |
| Yes | 352 (27.85%) | 289 (22.88%) | 449 (35.52%) |  |

**Revised Text**:

| **Parameters** | **Low** | **Medium** | **High** | **P-value** |
| --- | --- | --- | --- | --- |
|  | N=1264 | N=1263 | N=1264 |  |
| Age, years | 74 [63-84] | 72 [61-82] | 73 [61-83] | 0.015* |
| BUN,mg/dl | 17.00 (13.00-22.00) | 16.00 (12.00-21.00) | 18.00(13.00-24.00) | <0.001 |
| Chloride ,mEq/L | 104.02 (4.15) | 103.72 (3.96) | 102.89 (4.40) | <0.001 |
| Serum creatinine，mg/dL | 0.90 (0.80-1.20) | 0.90 (0.70-1.10) | 0.90 (0.80-1.20) | <0.001 |
| Blood glucose,mg/dL | 99.75 (28.57) | 114.91 (32.52) | 166.90 (71.41) | <0.001 |
| Hemoglobin,g/dL | 12.37 (1.88) | 12.69 (1.92) | 12.34 (2.11) | <0.001 |
| HBA1C% | 6.57 (1.63) | 6.07 (1.23) | 6.28 (1.44) | <0.001 |
| Bicarbonate,mEq/L | 25.28 (3.45) | 24.99 (2.97) | 24.14 (3.44) | <0.001 |
| Potassium,mEq/L | 4.03 (0.47) | 4.04 (0.50) | 4.09 (0.58) | 0.004 |
| Platelet ,k/uL | 233.95 (83.39) | 228.40 (86.29) | 223.79 (89.85) | 0.013 |
| Total cholesterol,mg/dL | 167.74 (44.22) | 173.15 (43.73) | 167.23 (49.63) | 0.002 |
| Triglyceride,mg/dL | 110.00 (80.00-146.00) | 108.00 (81.00-147.00) | 114.05 (83.00-154.00) | 0.026 |
| White blood cell, k/uL | 7.40 (6.00-9.20) | 7.80 (6.30-9.90) | 9.00 (6.90-11.90) | <0.001 |
| Gender |  |  |  | 0.086 |
| Male | 604 (47.78%) | 659 (52.18%) | 635 (50.24%) |  |
| Female | 660 (52.22%) | 604 (47.82%) | 629 (49.76%) |  |
| BMI |  |  |  | 0.067 |
| <23.9 | 158 (12.50%) | 131 (10.37%) | 136 (10.76%) |  |
| 23.9-27.9 | 205 (16.22%) | 174 (13.78%) | 177 (14.00%) |  |
| >27.9 | 271 (21.44%) | 296 (23.44%) | 323 (25.55%) |  |
| Missing | 630 (49.84%) | 662 (52.41%) | 628 (49.68%) |  |
| Marital status |  |  |  | <0.001 |
| Married | 588 (46.52%) | 598 (47.35%) | 557 (44.07%) |  |
| Single | 270 (21.36%) | 279 (22.09%) | 298 (23.58%) |  |
| Widowed | 248 (19.62%) | 212 (16.79%) | 212 (16.77%) |  |
| Divorced | 101 (7.99%) | 88 (6.97%) | 61 (4.83%) |  |
| Missing | 57 (4.51%) | 86 (6.81%) | 136 (10.76%) |  |
| Race |  |  |  | <0.001 |
| White | 840 (66.46%) | 894 (70.78%) | 833 (65.90%) |  |
| Other | 164 (12.97%) | 189 (14.96%) | 247 (19.54%) |  |
| Black | 222 (17.56%) | 145 (11.48%) | 157 (12.42%) |  |
| Asian | 38 (3.01%) | 35 (2.77%) | 27 (2.14%) |  |
| Albumin,g/dl |  |  |  | <0.001 |
| <3.8 | 261 (20.65%) | 207 (16.39%) | 292 (23.10%) |  |
| >=3.8 | 247 (19.54%) | 325 (25.73%) | 298 (23.58%) |  |
| Missing | 756 (59.81%) | 731 (57.88%) | 674 (53.32%) |  |
| ALT,iu/l |  |  |  | 0.004 |
| <18 | 360 (28.48%) | 351 (27.79%) | 327 (25.87%) |  |
| >=18 | 352 (27.85%) | 386 (30.56%) | 442 (34.97%) |  |
| Missing | 552 (43.67%) | 526 (41.65%) | 495 (39.16%) |  |
| AST,iu/l |  |  |  | <0.001 |
| <22 | 370 (29.27%) | 342 (27.08%) | 306 (24.21%) |  |
| >=22 | 348 (27.53%) | 397 (31.43%) | 468 (37.03%) |  |
| Missing | 546 (43.20%) | 524 (41.49%) | 490 (38.77%) |  |
| Creatine kinase,iu/l |  |  |  | <0.001 |
| 47-322 | 452 (35.76%) | 494 (39.11%) | 490 (38.77%) |  |
| <47 | 115 (9.10%) | 92 (7.28%) | 102 (8.07%) |  |
| >322 | 63 (4.98%) | 88 (6.97%) | 116 (9.18%) |  |
| Missing | 634 (50.16%) | 589 (46.63%) | 556 (43.99%) |  |
| Creatine kinase-MB,ng/mL |  |  |  | <0.001 |
| <3 | 271 (21.44%) | 237 (18.79%) | 233 (18.43%) |  |
| 3.0-10 | 317 (25.08%) | 354 (28.07%) | 339 (26.82%) |  |
| >10 | 19 (1.50%) | 36 (2.85%) | 75 (5.93%) |  |
| Missing | 657 (51.98%) | 634 (50.28%) | 617 (48.81%) |  |
| Lymphocyte, k/uL |  |  |  | <0.001 |
| <1.08 | 39 (3.09%) | 55 (4.35%) | 95 (7.52%) |  |
| 1.08-1.74 | 58 (4.59%) | 57 (4.51%) | 85 (6.72%) |  |
| >1.74 | 62 (4.91%) | 51 (4.04%) | 75 (5.93%) |  |
| Missing | 1105 (87.42%) | 1100 (87.09%) | 1009 (79.83%) |  |
| Neutrophil,k/uL |  |  |  | <0.001 |
| <5.1 | 64 (5.06%) | 59 (4.67%) | 66 (5.22%) |  |
| 5.1-8.1 | 65 (5.14%) | 60 (4.75%) | 72 (5.70%) |  |
| >8.1 | 30 (2.37%) | 44 (3.48%) | 117 (9.26%) |  |
| Missing | 1105 (87.42%) | 1100 (87.09%) | 1009 (79.83%) |  |
| Total bilirubin,mg/dL |  |  |  | <0.001 |
| <0.4 | 164 (12.97%) | 133 (10.53%) | 150 (11.87%) |  |
| 0.4-0.7 | 371 (29.35%) | 360 (28.50%) | 372 (29.43%) |  |
| >0.7 | 121 (9.57%) | 188 (14.89%) | 198 (15.66%) |  |
| Missing | 608 (48.10%) | 582 (46.08%) | 544 (43.04%) |  |
| AF | 265 (20.97%) | 258 (20.43%) | 297 (23.50%) | 0.135 |
| Alcohol use | 13 (1.03%) | 22 (1.74%) | 12 (0.95%) | 0.14 |
| Anemia | 151 (11.95%) | 135 (10.69%) | 255 (20.17%) | <0.001 |
| Cancer | 90 (7.12%) | 92 (7.28%) | 101 (7.99%) | 0.676 |
| CAD | 339 (26.82%) | 295 (23.36%) | 382 (30.22%) | <0.001 |
| CKD | 103 (8.15%) | 95 (7.52%) | 105 (8.31%) | 0.744 |
| Diabetes mellitus | 412 (32.59%) | 329 (26.05%) | 516 (40.82%) | <0.001 |
| HF | 220 (17.41%) | 163 (12.91%) | 276 (21.84%) | <0.001 |
| Hypertension | 495 (39.16%) | 501 (39.67%) | 519 (41.06%) | 0.601 |
| Hyperlipidemia | 495 (39.16%) | 481 (38.08%) | 405 (32.04%) | <0.001 |
| RF | 64 (5.06%) | 73 (5.78%) | 166 (13.13%) | <0.001 |
| Tobacco use | 99 (7.83%) | 102 (8.08%) | 75 (5.93%) | 0.076 |
| Anticoagulant drugs | 156 (12.34%) | 158 (12.51%) | 184 (14.56%) | 0.186 |
| Antiplatelet drugs | 352 (27.85%) | 353 (27.95%) | 315 (24.92%) | 0.149 |
| Follow-up time for GCS ≤ 8,days | 3.67 (2.08-5.78) | 3.74 (2.33-6.04) | 4.78 (2.70-8.33) | <0.001 |
| GCS ≤ 8 |  |  |  | <0.001 |
| No | 1205 (95.33%) | 1191 (94.30%) | 1088 (86.08%) |  |
| Yes | 59 (4.67%) | 72 (5.70%) | 176 (13.92%) |  |
| Long-term follow-up time,days | 22.52 (3.09-520.34) | 11.35 (3.00-240.25) | 16.39 (4.23-199.28) | 0.005 |
| Long-term mortality |  |  |  | <0.001 |
| No | 912 (72.15%) | 974 (77.12%) | 815 (64.48%) |  |
| Yes | 352 (27.85%) | 289 (22.88%) | 449 (35.52%) |  |

2. The data underlying the findings must be made available without restrictions. Please specify where they can be accessed, or explain the reasons why they cannot be shared.

**Response:**

We sincerely appreciate your emphasis on data transparency. We have now comprehensively enhanced the Data Availability section with the following additions:

**Lines 376-381:**

Data Availability

The supplementary material includes the subset of the MIMIC database utilized to substantiate our findings. It is important to note that access to the MIMIC database necessitates the completion of relevant training and the acquisition of appropriate permissions. Comprehensive information regarding access to the MIMIC-IV database is available at <https://physionet.org/content/mimiciv/2.2/>.

3. In section 2.2 Patients, the authors repeat a sentence.

**Response:**

**Original Text (**section 2.2 Patients**, Lines 100-103)**:

The extracted data included demographic information such as age, sex, race, marital status, and body mass index (BMI). Demographic information included age, sex, race, marital status, and body mass index (BMI).

**Revised Text**:

Demographic information included age, sex, race, marital status, and body mass index (BMI).

4. In figure 2, specify the time unit.

**Response:**

**Original Figure (**figure 2**)**:

**Revised Figure (**figure 2**)**

Reviewer #2:

1. My main concern with this paper is that it is similar to one previously published by the first author, “Association of albumin-corrected anion gap with severe consciousness disorders and outcomes in ischemic stroke: a retrospective MIMIC analysis”, <https://doi.org/10.1038/s41598-024-76324-x.> The data, statistical tests, and figures are all the same, the only substantive difference being that this new paper uses SHR as the independent variable while the previous paper treats ACAG as the independent variable. The two papers appear to be parts of a single exploratory analysis, not distinct hypothesis-driven studies.

**Response:**

Thank you for your review and feedback on our manuscript. In response to your concerns about the similarity between this study and the work previously published by the first author, we would like to clarify the following:

In this study, we included a total of 3,791 patients, among whom 3,337 had missing values for the albumin-corrected anion gap (ACAG), resulting in a missing proportion of 88%. Consequently, ACAG value could not be included as a variable in our analysis. However, to address your concern regarding potential confounding factors, we calculated the E-value as you suggested, to account for unmeasured confounding factors, including the impact of ACAG on the outcomes. The E-value calculation indicated that the association between SHR and severe consciousness disorders remained significant after controlling for potential confounding factors. This analysis strengthens the robustness of our findings and supports the rationale for considering the SHR as an independent variable.

We would like to emphasize that although both studies utilized the same dataset and employed similar statistical methods, the explanatory variables and research questions explored are distinct. We have supplemented the discussion section with the relevant analysis of the E-value to clearly demonstrate its importance in validating our research conclusions.

We conducted an E-value analysis to investigate potential unmeasured confounding factors affecting the association between severe consciousness disorders (GCS ≤ 8) and acute ischemic stroke (AIS) patients. Based on the outcome variable type defined by the hazard ratio (HR), we inputted the observed estimate of 1.52 (representing the association strength between SHR and GCS ≤ 8) and set the null value for the true effect at 1. The results showed an E-value of 2.41, indicating that for unmeasured confounding to completely negate the observed association, the following conditions must be met simultaneously: the strength of the association between exposure and confounding (RR.eu) must be ≥ 1.82, and the strength of the association between confounding and outcome (RR.ud) must be ≥ 4.15.

The figure below further illustrates the nonlinear relationship between RR.eu (the association between exposure and confounding) and RR.ud (the relationship between confounding and outcome). The curve reveals that as RR.eu increased, the critical threshold for RR.ud decreased. The coordinates marked by the red points (RR.eu = 1.82, RR.ud = 4.15) correspond to the boundary conditions of the E-value, suggesting that unmeasured confounding must exert a synergistic effect on both exposure and outcome to overturn the current conclusions.

This analysis indicates that unless there are strong unmeasured confounding factors (with an association strength of ≥ 1.82 with exposure and ≥ 4.15 with outcome), the association between SHR and severe consciousness disorders exhibits statistical robustness. This finding supports the reliability of the SHR as an independent predictor of neurological deterioration in patients with AIS.

**Original Text (Lines 343-347)**:

Second, the study was based on the MIMIC database, which has a limited scope and does not include all variables that may influence the relationship between SHR and patient outcomes, such as lifestyle factors, socioeconomic status, and genetic predisposition.

**Revised Text**:

Second, the study was based on the MIMIC database, which has a limited scope and does not include all variables that may influence the relationship between SHR and patient outcomes, such as lifestyle factors, Albumin-Corrected Anion Gap[34],socioeconomic status, and genetic predisposition. However, we used E-value sensitivity analysis to quantify the potential implications of unmeasured confounders and found that an unmeasured confounder was unlikely to explain the entirety of the effect[35].

2. The reasons for running three different series of statistical tests (ANOVA/chi-squared tests on group means, Cox hazard models to test for low GCS event likelihood conditional on SHR, and a nonlinear RCS regression model) were not given. It seems that the Cox models are the most theoretically appropriate analysis to run, in which case the group-mean tests are superfluous. The Cox models are already nonlinear (with multiplicative exponential effects), so it’s not clear why another type of nonlinear test (RCS) should be run, or how to interpret the results of the two models in relation to each other.

**Response:**

Thank you for your detailed review and valuable comments on our research. We appreciate the time and effort you invested in evaluating our manuscript. From a theoretical perspective, the Cox model is a suitable method for this type of analysis, as it provides a relative estimate of the risk of event occurrence. However, the RCS model allows for a more detailed description of potential non-linear relationships.

In response to your suggestions, we will include explanations and references in the statistical analysis section to clarify our methodology and findings.

**Original Text (Lines 149-150)**:

RCS was used to explore the potential nonlinear relationships between SHR and outcomes of GCS ≤8 and long-term mortality.

**Revised Text**:

Moreover, RCS was utilized to examine the potential dose-response relationship between the SHR and outcomes, which was adjusted for multiple covariates, as previously mentioned[15].

3. The initial group mean tests (ANOVA/chi-squared, table 1) include not only the target dependent variable GCS, but the independent variable SHR (redundant) and many other variables which the paper refers to as “confounding variables” (line 141). If these are merely confounding variables which need to be controlled for in a statistical model, then putting them into the model as additional independent variables suffices. There is no point to running statistical tests on whether these variables themselves have means which vary with the primary independent variable of interest.

**Response:**

Thank you for your insightful comments ，In light of your feedback, we have revised our analysis by removing the redundant comparisons for the independent variable SHR, as well as certain confounding variables.

**Original Text (line 177: Table 1. Baseline characteristics by SHR tertiles (N=3791))：**

| **Parameters** | **Low** | **Medium** | **High** | **P-value** |
| --- | --- | --- | --- | --- |
|  | N=1264 | N=1263 | N=1264 |  |
| Age, years | 74 [63-84] | 72 [61-82] | 73 [61-83] | 0.015 |
| BUN,mg/dl | 17.00 (13.00-22.00) | 16.00 (12.00-21.00) | 18.00(13.00-24.00) | <0.001 |
| Chloride,mEq/L | 104.02 (4.15) | 103.72 (3.96) | 102.89 (4.40) | <0.001 |
| SCR,mg/dL | 0.90 (0.80-1.20) | 0.90 (0.70-1.10) | 0.90 (0.80-1.20) | <0.001 |
| GLU,mg/dL | 99.75 (28.57) | 114.91 (32.52) | 166.90 (71.41) | <0.001 |
| HB,g/dL | 12.37 (1.88) | 12.69 (1.92) | 12.34 (2.11) | <0.001 |
| HBA1C% | 6.57 (1.63) | 6.07 (1.23) | 6.28 (1.44) | <0.001 |
| HCO3,mEq/L | 25.28 (3.45) | 24.99 (2.97) | 24.14 (3.44) | <0.001 |
| Potassium,mEq/L | 4.03 (0.47) | 4.04 (0.50) | 4.09 (0.58) | 0.004 |
| Platelet ,k/uL | 233.95 (83.39) | 228.40 (86.29) | 223.79 (89.85) | 0.013 |
| TC,mg/dL | 167.74 (44.22) | 173.15 (43.73) | 167.23 (49.63) | 0.002 |
| Triglyceride,mg/dL | 110.00 (80.00-146.00) | 108.00 (81.00-147.00) | 114.05 (83.00-154.00) | 0.026 |
| WBC, k/uL | 7.40 (6.00-9.20) | 7.80 (6.30-9.90) | 9.00 (6.90-11.90) | <0.001 |
| SHR, mg/dl | 0.72 (0.10) | 0.90 (0.05) | 1.24 (0.28) | <0.001 |
| Gender |  |  |  | 0.086 |
| Male | 604 (47.78%) | 659 (52.18%) | 635 (50.24%) |  |
| Female | 660 (52.22%) | 604 (47.82%) | 629 (49.76%) |  |
| BMI |  |  |  | 0.067 |
| <23.9 | 158 (12.50%) | 131 (10.37%) | 136 (10.76%) |  |
| 23.9-27.9 | 205 (16.22%) | 174 (13.78%) | 177 (14.00%) |  |
| >27.9 | 271 (21.44%) | 296 (23.44%) | 323 (25.55%) |  |
| Missing | 630 (49.84%) | 662 (52.41%) | 628 (49.68%) |  |
| Marital status |  |  |  | <0.001 |
| Married | 588 (46.52%) | 598 (47.35%) | 557 (44.07%) |  |
| Single | 270 (21.36%) | 279 (22.09%) | 298 (23.58%) |  |
| Widowed | 248 (19.62%) | 212 (16.79%) | 212 (16.77%) |  |
| Divorced | 101 (7.99%) | 88 (6.97%) | 61 (4.83%) |  |
| Missing | 57 (4.51%) | 86 (6.81%) | 136 (10.76%) |  |
| Race |  |  |  | <0.001 |
| White | 840 (66.46%) | 894 (70.78%) | 833 (65.90%) |  |
| Other | 164 (12.97%) | 189 (14.96%) | 247 (19.54%) |  |
| Black | 222 (17.56%) | 145 (11.48%) | 157 (12.42%) |  |
| Asian | 38 (3.01%) | 35 (2.77%) | 27 (2.14%) |  |
| ALB,g/dl |  |  |  | <0.001 |
| <3.8 | 261 (20.65%) | 207 (16.39%) | 292 (23.10%) |  |
| >=3.8 | 247 (19.54%) | 325 (25.73%) | 298 (23.58%) |  |
| Missing | 756 (59.81%) | 731 (57.88%) | 674 (53.32%) |  |
| ALT,iu/l |  |  |  | 0.004 |
| <18 | 360 (28.48%) | 351 (27.79%) | 327 (25.87%) |  |
| >=18 | 352 (27.85%) | 386 (30.56%) | 442 (34.97%) |  |
| Missing | 552 (43.67%) | 526 (41.65%) | 495 (39.16%) |  |
| AST,iu/l |  |  |  | <0.001 |
| <22 | 370 (29.27%) | 342 (27.08%) | 306 (24.21%) |  |
| >=22 | 348 (27.53%) | 397 (31.43%) | 468 (37.03%) |  |
| Missing | 546 (43.20%) | 524 (41.49%) | 490 (38.77%) |  |
| CK,iu/l |  |  |  | <0.001 |
| 47-322 | 452 (35.76%) | 494 (39.11%) | 490 (38.77%) |  |
| <47 | 115 (9.10%) | 92 (7.28%) | 102 (8.07%) |  |
| >322 | 63 (4.98%) | 88 (6.97%) | 116 (9.18%) |  |
| Missing | 634 (50.16%) | 589 (46.63%) | 556 (43.99%) |  |
| CKMB,ng/mL |  |  |  | <0.001 |
| <3 | 271 (21.44%) | 237 (18.79%) | 233 (18.43%) |  |
| 3.0-10 | 317 (25.08%) | 354 (28.07%) | 339 (26.82%) |  |
| >10 | 19 (1.50%) | 36 (2.85%) | 75 (5.93%) |  |
| Missing | 657 (51.98%) | 634 (50.28%) | 617 (48.81%) |  |
| Lymphocyte, k/uL |  |  |  | <0.001 |
| <1.08 | 39 (3.09%) | 55 (4.35%) | 95 (7.52%) |  |
| 1.08-1.74 | 58 (4.59%) | 57 (4.51%) | 85 (6.72%) |  |
| >1.74 | 62 (4.91%) | 51 (4.04%) | 75 (5.93%) |  |
| Missing | 1105 (87.42%) | 1100 (87.09%) | 1009 (79.83%) |  |
| Neutrophil,k/uL |  |  |  | <0.001 |
| <5.1 | 64 (5.06%) | 59 (4.67%) | 66 (5.22%) |  |
| 5.1-8.1 | 65 (5.14%) | 60 (4.75%) | 72 (5.70%) |  |
| >8.1 | 30 (2.37%) | 44 (3.48%) | 117 (9.26%) |  |
| Missing | 1105 (87.42%) | 1100 (87.09%) | 1009 (79.83%) |  |
| TBIL,mg/dL |  |  |  | <0.001 |
| <0.4 | 164 (12.97%) | 133 (10.53%) | 150 (11.87%) |  |
| 0.4-0.7 | 371 (29.35%) | 360 (28.50%) | 372 (29.43%) |  |
| >0.7 | 121 (9.57%) | 188 (14.89%) | 198 (15.66%) |  |
| Missing | 608 (48.10%) | 582 (46.08%) | 544 (43.04%) |  |
| AF | 265 (20.97%) | 258 (20.43%) | 297 (23.50%) | 0.135 |
| Alcohol use | 13 (1.03%) | 22 (1.74%) | 12 (0.95%) | 0.14 |
| Anemia | 151 (11.95%) | 135 (10.69%) | 255 (20.17%) | <0.001 |
| Cancer | 90 (7.12%) | 92 (7.28%) | 101 (7.99%) | 0.676 |
| CAD | 339 (26.82%) | 295 (23.36%) | 382 (30.22%) | <0.001 |
| CKD | 103 (8.15%) | 95 (7.52%) | 105 (8.31%) | 0.744 |
| DM | 412 (32.59%) | 329 (26.05%) | 516 (40.82%) | <0.001 |
| HF | 220 (17.41%) | 163 (12.91%) | 276 (21.84%) | <0.001 |
| HBP | 495 (39.16%) | 501 (39.67%) | 519 (41.06%) | 0.601 |
| HL | 495 (39.16%) | 481 (38.08%) | 405 (32.04%) | <0.001 |
| RF | 64 (5.06%) | 73 (5.78%) | 166 (13.13%) | <0.001 |
| Tobacco use | 99 (7.83%) | 102 (8.08%) | 75 (5.93%) | 0.076 |
| Anticoagulant drugs | 156 (12.34%) | 158 (12.51%) | 184 (14.56%) | 0.186 |
| Antiplatelet drugs | 352 (27.85%) | 353 (27.95%) | 315 (24.92%) | 0.149 |
| Follow-up time for GCS ≤ 8,days | 3.67 (2.08-5.78) | 3.74 (2.33-6.04) | 4.78 (2.70-8.33) | <0.001 |
| GCS ≤ 8 |  |  |  | <0.001 |
| No | 1205 (95.33%) | 1191 (94.30%) | 1088 (86.08%) |  |
| Yes | 59 (4.67%) | 72 (5.70%) | 176 (13.92%) |  |
| Long-term follow-up time,days | 22.52 (3.09-520.34) | 11.35 (3.00-240.25) | 16.39 (4.23-199.28) | 0.005 |
| Long-term mortality |  |  |  | <0.001 |
| No | 912 (72.15%) | 974 (77.12%) | 815 (64.48%) |  |
| Yes | 352 (27.85%) | 289 (22.88%) | 449 (35.52%) |  |

**Revised Text**:

| **Parameters** | **Low** | **Medium** | **High** | **P-value** |
| --- | --- | --- | --- | --- |
|  | N=1264 | N=1263 | N=1264 |  |
| Age, years | 74 [63-84] | 72 [61-82] | 73 [61-83] | 0.015* |
| BUN,mg/dl | 17.00 (13.00-22.00) | 16.00 (12.00-21.00) | 18.00(13.00-24.00) | <0.001 |
| Chloride,mEq/L | 104.02 (4.15) | 103.72 (3.96) | 102.89 (4.40) | <0.001 |
| Creatine kinase,mg/dL | 0.90 (0.80-1.20) | 0.90 (0.70-1.10) | 0.90 (0.80-1.20) | <0.001 |
| Blood glucose,mg/dL | 99.75 (28.57) | 114.91 (32.52) | 166.90 (71.41) | <0.001 |
| Hemoglobin,g/dL | 12.37 (1.88) | 12.69 (1.92) | 12.34 (2.11) | <0.001 |
| HBA1C% | 6.57 (1.63) | 6.07 (1.23) | 6.28 (1.44) | <0.001 |
| Bicarbonate,mEq/L | 25.28 (3.45) | 24.99 (2.97) | 24.14 (3.44) | <0.001 |
| Potassium,mEq/L | 4.03 (0.47) | 4.04 (0.50) | 4.09 (0.58) | 0.004 |
| Platelet ,k/uL | 233.95 (83.39) | 228.40 (86.29) | 223.79 (89.85) | 0.013 |
| Total cholesterol,mg/dL | 167.74 (44.22) | 173.15 (43.73) | 167.23 (49.63) | 0.002 |
| Triglyceride,mg/dL | 110.00 (80.00-146.00) | 108.00 (81.00-147.00) | 114.05 (83.00-154.00) | 0.026 |
| white blood cell, k/uL | 7.40 (6.00-9.20) | 7.80 (6.30-9.90) | 9.00 (6.90-11.90) | <0.001 |
| Gender |  |  |  | 0.086 |
| Male | 604 (47.78%) | 659 (52.18%) | 635 (50.24%) |  |
| Female | 660 (52.22%) | 604 (47.82%) | 629 (49.76%) |  |
| BMI |  |  |  | 0.067 |
| <23.9 | 158 (12.50%) | 131 (10.37%) | 136 (10.76%) |  |
| 23.9-27.9 | 205 (16.22%) | 174 (13.78%) | 177 (14.00%) |  |
| >27.9 | 271 (21.44%) | 296 (23.44%) | 323 (25.55%) |  |
| Missing | 630 (49.84%) | 662 (52.41%) | 628 (49.68%) |  |
| Marital status |  |  |  | <0.001 |
| Married | 588 (46.52%) | 598 (47.35%) | 557 (44.07%) |  |
| Single | 270 (21.36%) | 279 (22.09%) | 298 (23.58%) |  |
| Widowed | 248 (19.62%) | 212 (16.79%) | 212 (16.77%) |  |
| Divorced | 101 (7.99%) | 88 (6.97%) | 61 (4.83%) |  |
| Missing | 57 (4.51%) | 86 (6.81%) | 136 (10.76%) |  |
| Race |  |  |  | <0.001 |
| White | 840 (66.46%) | 894 (70.78%) | 833 (65.90%) |  |
| Other | 164 (12.97%) | 189 (14.96%) | 247 (19.54%) |  |
| Black | 222 (17.56%) | 145 (11.48%) | 157 (12.42%) |  |
| Asian | 38 (3.01%) | 35 (2.77%) | 27 (2.14%) |  |
| albumin,g/dl |  |  |  | <0.001 |
| <3.8 | 261 (20.65%) | 207 (16.39%) | 292 (23.10%) |  |
| >=3.8 | 247 (19.54%) | 325 (25.73%) | 298 (23.58%) |  |
| Missing | 756 (59.81%) | 731 (57.88%) | 674 (53.32%) |  |
| ALT,iu/l |  |  |  | 0.004 |
| <18 | 360 (28.48%) | 351 (27.79%) | 327 (25.87%) |  |
| >=18 | 352 (27.85%) | 386 (30.56%) | 442 (34.97%) |  |
| Missing | 552 (43.67%) | 526 (41.65%) | 495 (39.16%) |  |
| AST,iu/l |  |  |  | <0.001 |
| <22 | 370 (29.27%) | 342 (27.08%) | 306 (24.21%) |  |
| >=22 | 348 (27.53%) | 397 (31.43%) | 468 (37.03%) |  |
| Missing | 546 (43.20%) | 524 (41.49%) | 490 (38.77%) |  |
| CK creatine kinase,iu/l |  |  |  | <0.001 |
| 47-322 | 452 (35.76%) | 494 (39.11%) | 490 (38.77%) |  |
| <47 | 115 (9.10%) | 92 (7.28%) | 102 (8.07%) |  |
| >322 | 63 (4.98%) | 88 (6.97%) | 116 (9.18%) |  |
| Missing | 634 (50.16%) | 589 (46.63%) | 556 (43.99%) |  |
| creatine kinase-MB,ng/mL |  |  |  | <0.001 |
| <3 | 271 (21.44%) | 237 (18.79%) | 233 (18.43%) |  |
| 3.0-10 | 317 (25.08%) | 354 (28.07%) | 339 (26.82%) |  |
| >10 | 19 (1.50%) | 36 (2.85%) | 75 (5.93%) |  |
| Missing | 657 (51.98%) | 634 (50.28%) | 617 (48.81%) |  |
| Lymphocyte, k/uL |  |  |  | <0.001 |
| <1.08 | 39 (3.09%) | 55 (4.35%) | 95 (7.52%) |  |
| 1.08-1.74 | 58 (4.59%) | 57 (4.51%) | 85 (6.72%) |  |
| >1.74 | 62 (4.91%) | 51 (4.04%) | 75 (5.93%) |  |
| Missing | 1105 (87.42%) | 1100 (87.09%) | 1009 (79.83%) |  |
| Neutrophil,k/uL |  |  |  | <0.001 |
| <5.1 | 64 (5.06%) | 59 (4.67%) | 66 (5.22%) |  |
| 5.1-8.1 | 65 (5.14%) | 60 (4.75%) | 72 (5.70%) |  |
| >8.1 | 30 (2.37%) | 44 (3.48%) | 117 (9.26%) |  |
| Missing | 1105 (87.42%) | 1100 (87.09%) | 1009 (79.83%) |  |
| total bilirubin,mg/dL |  |  |  | <0.001 |
| <0.4 | 164 (12.97%) | 133 (10.53%) | 150 (11.87%) |  |
| 0.4-0.7 | 371 (29.35%) | 360 (28.50%) | 372 (29.43%) |  |
| >0.7 | 121 (9.57%) | 188 (14.89%) | 198 (15.66%) |  |
| Missing | 608 (48.10%) | 582 (46.08%) | 544 (43.04%) |  |
| AF | 265 (20.97%) | 258 (20.43%) | 297 (23.50%) | 0.135 |
| Alcohol use | 13 (1.03%) | 22 (1.74%) | 12 (0.95%) | 0.14 |
| Anemia | 151 (11.95%) | 135 (10.69%) | 255 (20.17%) | <0.001 |
| Cancer | 90 (7.12%) | 92 (7.28%) | 101 (7.99%) | 0.676 |
| CAD | 339 (26.82%) | 295 (23.36%) | 382 (30.22%) | <0.001 |
| CKD | 103 (8.15%) | 95 (7.52%) | 105 (8.31%) | 0.744 |
| diabetes mellitus | 412 (32.59%) | 329 (26.05%) | 516 (40.82%) | <0.001 |
| HF | 220 (17.41%) | 163 (12.91%) | 276 (21.84%) | <0.001 |
| Hypertension | 495 (39.16%) | 501 (39.67%) | 519 (41.06%) | 0.601 |
| Hyperlipidemia | 495 (39.16%) | 481 (38.08%) | 405 (32.04%) | <0.001 |
| RF | 64 (5.06%) | 73 (5.78%) | 166 (13.13%) | <0.001 |
| Tobacco use | 99 (7.83%) | 102 (8.08%) | 75 (5.93%) | 0.076 |

4. There is no discussion of any multiple-comparisons corrections, but such corrections seem to be needed. I counted 104 tests reported in the paper, many with a p-value evidently between 0.001 and 0.0001, which means many of those p-values are not likely to remain above a significance threshold of 0.05 after correction (because 0.001 x 100 = 0.1). In addition, the fact that the analysis here so closely parallels the one published in the ACAG paper raises the worry that the actual number of tests run on the data is higher than reported here.

**Response:**

We sincerely appreciate the reviewer for pointing out these errors. Due to oversight, there were inaccuracies in the description of the statistical analysis methods, and we used the Kruskal-Wallis test to compare groups of continuous variables. Therefore, we re-conducted the Shapiro-Wilk test on continuous variables, corrected the description of age, and revised the relevant sections of the statistical analysis accordingly.

**1) Original Text (Lines 20-23):**

**Methods**

This retrospective cohort study based on the MIMIC database included 3,791 AIS patients with a mean age of 71.10 years, of whom 49.93% were female.

**Revised Text**:

**Methods**

This retrospective cohort study based on the MIMIC database.

**2) Original Text (Lines 158-159):**

A total of 3,791 patients were included in this study (Fig 1), with a mean age of 71.10 years.

**Revised Text**:

A total of 3,791 patients were included in this study (Fig 1), with a median age of 73 years (interquartile range [IQR]: 61–83)

3) **Original Text (line 177,** Table 1. Baseline characteristics by SHR tertiles (N=3791)**):**

| **Parameters** | **Low** | **Medium** | **High** | **P-value** |
| --- | --- | --- | --- | --- |
|  | N=1264 | N=1263 | N=1264 |  |
| Age, years | 71.96 (14.75) | 70.42 (14.78) | 70.91 (14.88) | 0.028 |

**Revised Text**:

| **Parameters** | **Low** | **Medium** | **High** | **P-value** |
| --- | --- | --- | --- | --- |
|  | N=1264 | N=1263 | N=1264 |  |
| Age, years | 74 [63-84] | 72 [61-82] | 73 [61-83] | 0.015 |

4) We conducted the Shapiro-Wilk test on the data and found that all continuous variables were not normally distributed (Table S2). Therefore, we used Kruskal-Wallis test for continuous variables and corrected the tables accordingly. Additionally, as per your request, we performed Dunn's test with Bonferroni correction for continuous variables(Table **S3**), and we applied Chi-square tests or Fisher's exact tests with FDR correction for categorical variables(Table **S4**).

**S2 Table：Shapiro-Wilk test**

| Variable | W Statistic | P Value |
| --- | --- | --- |
| Age | 0.9664 | < 2.2e-16 |
| BUN | 0.7537 | < 2.2e-16 |
| Chloride | 0.9766 | < 2.2e-16 |
| Creatine kinase | 0.4051 | < 2.2e-16 |
| Blood glucose | 0.724 | < 2.2e-16 |
| Hemoglobin | 0.9899 | 7.86E-16 |
| HbA1c | 0.7401 | < 2.2e-16 |
| Bicarbonate | 0.9812 | < 2.2e-16 |
| Potassium | 0.9263 | < 2.2e-16 |
| PLT | 0.8725 | < 2.2e-16 |
| Total cholesterol | 0.9549 | < 2.2e-16 |
| Triglyceride | 0.5332 | < 2.2e-16 |
| White blood cell | 0.5652 | < 2.2e-16 |

**Results:**

The Shapiro-Wilk test results indicated that none of the continuous variables in this study were normally distributed, as all p-values were significantly below the conventional alpha level of 0.05 (**S2** Table).

**S3 Table：Statistical Analysis of Continuous Variables Using Dunn's Test with Bonferroni Correction Across SHR Tertiles**

| Variable | High vs. Low | High vs. Medium | Low vs. Medium |
| --- | --- | --- | --- |
| Age | Z = -1.86, ns | Z = 0.99, ns | Z = 2.86, ** |
| Blood urea nitrogen (BUN) | Z = 3.02, ** | Z = 5.07, *** | Z = 2.05, ns |
| Chloride | Z = -6.98, ** | Z = -5.49, *** | Z = 1.49, ns |
| Creatine kinase | Z = 1.40, ns | Z = 3.75, *** | Z = 2.35, * |
| Blood glucose | Z = 40.00, ** | Z = 24.07, *** | Z = -15.93, *** |
| Hemoglobin | Z = 0.34, ns | Z = -4.33, *** | Z = -4.67, *** |
| HbA1c | Z = -7.68, ** | Z = 3.45, *** | Z = 11.13, *** |
| Bicarbonate | Z = -8.71, ** | Z = -6.38, *** | Z = 2.34, * |
| Potassium | Z = 1.55, ns | Z = 1.42, ns | Z = -0.14, ns |
| Platelet | Z = -3.61, ** | Z = -1.55, ns | Z = 2.06, ns |
| Total cholesterol | Z = -0.68, ns | Z = -4.37, *** | Z = -3.69, *** |
| Triglyceride | Z = 2.40, * | Z = 2.28, * | Z = -0.12, ns |
| White blood cell | Z = 12.74, ** | Z = 9.01, *** | Z = -3.73, *** |
|  | | | |

Statistical comparisons were performed using the Mann-Whitney U test. P adj < 0.001 is indicated by "***"; P adj < 0.01 is indicated by "**"; P adj < 0.05 is indicated by "*"; ns = not significant.

**Results:**

The results of this study indicate that after applying Bonferroni correction, several continuous variables showed significant differences across the SHR tertiles, particularly with respect to blood glucose, HbA1c, and White blood cell count, which demonstrated significant variations between the different groups(**S3**Table).

**S4 Table:** **Statistical Evaluation of Categorical Variables by SHR Tertiles Using Chi-Square and Fisher's Exact Tests with FDR Correction**

| **Variable** | **Low vs Medium** | **Low vs High** | **Medium vs High** |
| --- | --- | --- | --- |
| Gender | p=0.030 * | p=0.233 ns | p=0.349 ns |
| BMI | p=0.073 ns | p=0.041 * | p=0.533 ns |
| Marital Status | p=0.043 * | p=0.000 *** | p=0.001 ** |
| Race | p=0.000 *** | p=0.000 *** | p=0.010 ** |
| Albumin | p=0.000 *** | p=0.004 ** | p=0.000 *** |
| ALT | p=0.316 ns | p=0.001 *** | p=0.061 ns |
| AST | p=0.092 ns | p=0.000 *** | p=0.011 * |
| Creatine Kinase | p=0.017 * | p=0.000 *** | p=0.149 ns |
| Creatine Kinase-MB | p=0.019 * | p=0.000 *** | p=0.002 ** |
| Lymphocyte | p=0.282 ns | p=0.000 *** | p=0.000 *** |
| Neutrophil | p=0.382 ns | p=0.000 *** | p=0.000 *** |
| Total Bilirubin | p=0.000 *** | p=0.000 *** | p=0.430 ns |
| AF | p=0.776 ns | p=0.138 ns | p=0.069 ns |
| Anemia | p=0.350 ns | p=0.000 *** | p=0.000 *** |
| Cancer | p=0.934 ns | p=0.452 ns | p=0.553 ns |
| CHD | p=0.050 * | p=0.064 ns | p=0.000 *** |
| CKD | p=0.608 ns | p=0.942 ns | p=0.511 ns |
| DM | p=0.000 *** | p=0.000 *** | p=0.000 *** |
| HF | p=0.002 ** | p=0.006 ** | p=0.000 *** |
| Hypertension | p=0.606 ns | p=0.000 *** | p=0.002 ** |
| RF | p=0.479 ns | p=0.000 *** | p=0.000 *** |
| Tobacco Use | p=0.878 ns | p=0.071 ns | p=0.042 * |

All statistical comparisons were conducted using chi-square tests or Fisher's exact tests (depending on the data characteristics). P adj < 0.001 is indicated by "***"; P adj < 0.01 is indicated by "**"; P adj < 0.05 is indicated by "*"; ns = not significant.

**Results:**

The comparison of multiple categorical variables across SHR tertiles showed significant differences, particularly concerning diabetes, heart failure, and albumin levels(**S4** Table)..

**5) Original Text (Lines 163-173):**

The high SHR group had a significantly higher GCS ≤8 (13.92%) and long-term mortality (35.52%) than the low (4.67% and 27.85 %, respectively) and medium (5.70% and 22.88 %, respectively) SHR groups (p < 0.001). Additionally, patients in the High SHR group had significantly higher BUN and WBC counts than the other two groups (p < 0.001). Moreover, the prevalence of comorbidities, such as DM (40.82%), anemia (20.17%), CAD (30.22%), HF (21.84%), and RF (13.13%), was significantly higher in the high SHR group than in the low and medium SHR groups (p < 0.001)(Table 1).

**Revised Text**:

The high SHR group exhibited significantly higher levels of blood urea nitrogen (BUN) and white blood cell (WBC) counts compared to both the low SHR group and the medium SHR group (p < 0.001)(Table1,S3). In terms of comorbidities, the prevalence of diabetes mellitus (DM) in the high SHR group was 40.82%, which was significantly greater than that in the low SHR group (p < 0.001) and the medium SHR group (p < 0.001). Additionally, the prevalence of heart failure (HF) was 21.84%, similarly being significantly higher than that in the low SHR group (p < 0.001) and the medium SHR group (p = 0.006 )( Table1,S4)

5. The paper is not clear in many areas. For example, line 131 is a description of table 1 that should go in a caption, not part of the statistical analysis. This paragraph makes no reference to table 1, and I did not understand it until I saw table 1. It would be helpful if the author’s published the R code they used, as someone could only reconstruct the nuts-and-bolts of their analysis with considerable effort.

**Response:**

**Original Text (Lines 131-136):**

Continuous variables were expressed as mean ± standard deviation (SD) or median with interquartile range (IQR), while categorical variables were presented as counts and percentages. Patients were divided into tertiles based on their SHR. Differences in continuous variables were compared using one-way analysis of variance (ANOVA), and differences in categorical variables were assessed using chi-squared tests.

**Revised Text**:

Continuous variables were expressed as mean ± standard deviation (SD) or median with interquartile range (IQR), while categorical variables were presented as counts and percentages. Differences in continuous variables were compared using Wilcoxon or Kruskal-Wallis tests, and differences in categorical variables were assessed using chi-squared tests.

2) Thank you for your valuable feedback regarding our manuscript. We recognize that providing the R code used in our analysis will greatly assist other researchers in reconstructing our work. Therefore, we have decided to include the relevant R code in the revised manuscript to ensure that readers can access and replicate our analytical process more easily (**R code.PDF)**.

6. Some of the references have issues. For example, the first line of the paper says that AIS “is the leading cause of death and long-term disability worldwide”, but this is not true, and the reference given contradicts it.

**Response:**

We appreciate your feedback. We have corrected the inaccuracies in the citations and descriptions. The specific modifications are as follows.

**Original Text (Lines 51-52):**

Acute ischemic stroke (AIS) is the leading cause of death and long-term disability worldwide[1].

**Revised Text**:

Acute ischemic stroke (AIS) is a significant cause of mortality and long-term disability globally, accounting for a large proportion of stroke cases, and remains the second leading cause of death worldwide[1].
